# Supplementary material for: Modeling glioblastoma heterogeneity as a dynamic network of cell states
Source: Mol Syst Biol. 2021 Sep 16;17(9):e10105. doi: 10.15252/msb.202010105 (PMC8444284; doi:10.15252/msb.202010105)
Supplement: Supplementary file 5 — Source Data for Figure 3 [file MSB-17-e10105-s001.zip › Figure3A_sourcedata/GSEA_3065/nosigdb_state4.GseaPreranked.1622718430994/FELDMAN_2020_G2-M.html]

Details for gene set FELDMAN\_2020\_G2-M[GSEA]

|  || Dataset | state4 |
| Phenotype | NoPhenotypeAvailable |
| Upregulated in class | na\_neg |
| GeneSet | FELDMAN\_2020\_G2-M |
| Enrichment Score (ES) | -0.67720926 |
| Normalized Enrichment Score (NES) | -2.7109382 |
| Nominal p-value | 0.0 |
| FDR q-value | 0.0 |
| FWER p-Value | 0.0 |
Table: GSEA Results Summary

  

Fig 1: Enrichment plot: FELDMAN\_2020\_G2-M      
 Profile of the Running ES Score & Positions of GeneSet Members on the Rank Ordered List

  

| PROBE | GENE SYMBOL | GENE\_TITLE | RANK IN GENE LIST | RANK METRIC SCORE | RUNNING ES | CORE ENRICHMENT || 1 | SAT1 |  |  | 4 | 0.669 | 0.0092 | No |
| 2 | FABP7 |  |  | 5 | 0.656 | 0.0189 | No |
| 3 | S100B |  |  | 6 | 0.630 | 0.0282 | No |
| 4 | FTH1 |  |  | 8 | 0.512 | 0.0356 | No |
| 5 | BEX1 |  |  | 11 | 0.473 | 0.0422 | No |
| 6 | EEF1A1 |  |  | 12 | 0.471 | 0.0492 | No |
| 7 | FTL |  |  | 13 | 0.466 | 0.0561 | No |
| 8 | METRN |  |  | 14 | 0.462 | 0.0629 | No |
| 9 | SOX4 |  |  | 18 | 0.402 | 0.0683 | No |
| 10 | COMMD6 |  |  | 36 | 0.334 | 0.0704 | No |
| 11 | OST4 |  |  | 37 | 0.325 | 0.0752 | No |
| 12 | C4orf3 |  |  | 43 | 0.308 | 0.0789 | No |
| 13 | C4orf48 |  |  | 59 | 0.291 | 0.0807 | No |
| 14 | RPL34 |  |  | 69 | 0.278 | 0.0833 | No |
| 15 | CLU |  |  | 95 | 0.252 | 0.0828 | No |
| 16 | TTYH1 |  |  | 100 | 0.249 | 0.0858 | No |
| 17 | PCSK1N |  |  | 172 | 0.212 | 0.0771 | No |
| 18 | BEX4 |  |  | 220 | 0.194 | 0.0721 | No |
| 19 | GLIPR2 |  |  | 312 | 0.172 | 0.0594 | No |
| 20 | CDKN1A |  |  | 319 | 0.171 | 0.0610 | No |
| 21 | PLP1 |  |  | 330 | 0.169 | 0.0618 | No |
| 22 | ATP6V0E1 |  |  | 338 | 0.168 | 0.0631 | No |
| 23 | GPM6B |  |  | 346 | 0.166 | 0.0644 | No |
| 24 | TP53I3 |  |  | 353 | 0.164 | 0.0658 | No |
| 25 | PRDX1 |  |  | 416 | 0.149 | 0.0576 | No |
| 26 | TCEAL8 |  |  | 440 | 0.145 | 0.0559 | No |
| 27 | H3F3B |  |  | 447 | 0.144 | 0.0570 | No |
| 28 | RPL31 |  |  | 484 | 0.139 | 0.0531 | No |
| 29 | DDIT4 |  |  | 493 | 0.138 | 0.0538 | No |
| 30 | SPARC |  |  | 539 | 0.132 | 0.0482 | No |
| 31 | IFITM3 |  |  | 546 | 0.132 | 0.0491 | No |
| 32 | CALM3 |  |  | 570 | 0.129 | 0.0472 | No |
| 33 | PSAP |  |  | 596 | 0.126 | 0.0449 | No |
| 34 | FAM162A |  |  | 660 | 0.119 | 0.0361 | No |
| 35 | PLD3 |  |  | 666 | 0.118 | 0.0370 | No |
| 36 | EEF2 |  |  | 729 | 0.110 | 0.0283 | No |
| 37 | CST3 |  |  | 792 | 0.104 | 0.0195 | No |
| 38 | SHISA4 |  |  | 848 | 0.099 | 0.0117 | No |
| 39 | IGFBP5 |  |  | 901 | 0.096 | 0.0045 | No |
| 40 | RBP1 |  |  | 1171 | 0.078 | -0.0393 | No |
| 41 | RAB13 |  |  | 1270 | 0.072 | -0.0547 | No |
| 42 | EIF4A2 |  |  | 1311 | 0.070 | -0.0603 | No |
| 43 | PDLIM4 |  |  | 1446 | 0.064 | -0.0817 | No |
| 44 | MT2A |  |  | 1542 | 0.060 | -0.0967 | No |
| 45 | PRCP |  |  | 1675 | 0.056 | -0.1180 | No |
| 46 | TM7SF2 |  |  | 1689 | 0.056 | -0.1193 | No |
| 47 | CCDC167 |  |  | 1711 | 0.055 | -0.1220 | No |
| 48 | PLIN3 |  |  | 1974 | 0.048 | -0.1651 | No |
| 49 | CBX1 |  |  | 2104 | 0.044 | -0.1860 | No |
| 50 | PDLIM7 |  |  | 2389 | 0.037 | -0.2329 | No |
| 51 | ISCA2 |  |  | 2477 | 0.035 | -0.2469 | No |
| 52 | CALD1 |  |  | 2682 | 0.031 | -0.2806 | No |
| 53 | CDKN2C |  |  | 2822 | 0.028 | -0.3034 | No |
| 54 | PSRC1 |  |  | 2990 | 0.025 | -0.3309 | No |
| 55 | MORF4L2 |  |  | 3509 | 0.012 | -0.4173 | No |
| 56 | SNRNP25 |  |  | 3634 | 0.008 | -0.4379 | No |
| 57 | TUBA1B |  |  | 3658 | 0.007 | -0.4417 | No |
| 58 | UBC |  |  | 3691 | 0.006 | -0.4469 | No |
| 59 | NUDCD2 |  |  | 3733 | 0.004 | -0.4537 | No |
| 60 | SSNA1 |  |  | 3823 | -0.000 | -0.4686 | No |
| 61 | CALM2 |  |  | 3844 | -0.001 | -0.4719 | No |
| 62 | PPP1CC |  |  | 3904 | -0.004 | -0.4817 | No |
| 63 | CHCHD2 |  |  | 3928 | -0.006 | -0.4855 | No |
| 64 | HNRNPA1 |  |  | 4032 | -0.011 | -0.5025 | No |
| 65 | BTG1 |  |  | 4064 | -0.013 | -0.5075 | No |
| 66 | DCXR |  |  | 4188 | -0.021 | -0.5278 | No |
| 67 | MAD2L2 |  |  | 4387 | -0.035 | -0.5603 | No |
| 68 | MIAT |  |  | 4438 | -0.039 | -0.5681 | No |
| 69 | GLRX5 |  |  | 4552 | -0.048 | -0.5863 | No |
| 70 | RNF26 |  |  | 4647 | -0.055 | -0.6012 | No |
| 71 | G2E3 |  |  | 4722 | -0.062 | -0.6126 | No |
| 72 | MEST |  |  | 4739 | -0.063 | -0.6144 | No |
| 73 | EMC9 |  |  | 4794 | -0.068 | -0.6224 | No |
| 74 | TNFAIP8L1 |  |  | 4806 | -0.068 | -0.6233 | No |
| 75 | FOSL1 |  |  | 4845 | -0.072 | -0.6285 | No |
| 76 | HOXB4 |  |  | 4890 | -0.074 | -0.6348 | No |
| 77 | RHNO1 |  |  | 4893 | -0.075 | -0.6340 | No |
| 78 | CYCS |  |  | 4894 | -0.075 | -0.6329 | No |
| 79 | NUP35 |  |  | 4956 | -0.079 | -0.6420 | No |
| 80 | SCRG1 |  |  | 4970 | -0.080 | -0.6429 | No |
| 81 | VDAC3 |  |  | 4979 | -0.081 | -0.6431 | No |
| 82 | EIF5 |  |  | 4991 | -0.082 | -0.6437 | No |
| 83 | HSD17B11 |  |  | 5042 | -0.086 | -0.6508 | No |
| 84 | ITM2B |  |  | 5050 | -0.087 | -0.6507 | No |
| 85 | MRPL51 |  |  | 5057 | -0.088 | -0.6504 | No |
| 86 | HP1BP3 |  |  | 5074 | -0.089 | -0.6518 | No |
| 87 | LSM3 |  |  | 5075 | -0.089 | -0.6504 | No |
| 88 | TUBB6 |  |  | 5076 | -0.089 | -0.6491 | No |
| 89 | CDC42EP3 |  |  | 5087 | -0.091 | -0.6495 | No |
| 90 | GLIPR1 |  |  | 5091 | -0.091 | -0.6486 | No |
| 91 | SKA3 |  |  | 5136 | -0.095 | -0.6546 | No |
| 92 | GAS2L3 |  |  | 5152 | -0.097 | -0.6556 | No |
| 93 | BCL2L12 |  |  | 5219 | -0.103 | -0.6651 | No |
| 94 | HMGN1 |  |  | 5223 | -0.103 | -0.6641 | No |
| 95 | FGFBP3 |  |  | 5233 | -0.104 | -0.6641 | No |
| 96 | RNASEH2A |  |  | 5241 | -0.105 | -0.6637 | No |
| 97 | KIF20A |  |  | 5260 | -0.107 | -0.6652 | No |
| 98 | PTN |  |  | 5261 | -0.107 | -0.6636 | No |
| 99 | DCP2 |  |  | 5283 | -0.108 | -0.6655 | No |
| 100 | ILF2 |  |  | 5331 | -0.112 | -0.6717 | No |
| 101 | RHEB |  |  | 5346 | -0.113 | -0.6724 | No |
| 102 | TNC |  |  | 5359 | -0.114 | -0.6727 | No |
| 103 | TCP1 |  |  | 5367 | -0.115 | -0.6721 | No |
| 104 | CBX3 |  |  | 5377 | -0.116 | -0.6719 | No |
| 105 | H2AFV |  |  | 5381 | -0.117 | -0.6707 | No |
| 106 | FAM83D |  |  | 5384 | -0.117 | -0.6693 | No |
| 107 | HES6 |  |  | 5392 | -0.118 | -0.6688 | No |
| 108 | RBM8A |  |  | 5398 | -0.118 | -0.6679 | No |
| 109 | HNRNPA3 |  |  | 5455 | -0.126 | -0.6754 | Yes |
| 110 | STIP1 |  |  | 5458 | -0.126 | -0.6738 | Yes |
| 111 | LRR1 |  |  | 5459 | -0.126 | -0.6720 | Yes |
| 112 | KIF5B |  |  | 5464 | -0.127 | -0.6708 | Yes |
| 113 | TUBB |  |  | 5482 | -0.129 | -0.6717 | Yes |
| 114 | RAD21 |  |  | 5496 | -0.131 | -0.6719 | Yes |
| 115 | NEK2 |  |  | 5505 | -0.132 | -0.6713 | Yes |
| 116 | CCDC18 |  |  | 5519 | -0.133 | -0.6715 | Yes |
| 117 | CKAP5 |  |  | 5549 | -0.136 | -0.6744 | Yes |
| 118 | SPDL1 |  |  | 5560 | -0.137 | -0.6740 | Yes |
| 119 | MAP3K7CL |  |  | 5565 | -0.138 | -0.6727 | Yes |
| 120 | DEPDC1B |  |  | 5570 | -0.138 | -0.6713 | Yes |
| 121 | BUB3 |  |  | 5583 | -0.141 | -0.6712 | Yes |
| 122 | HMGB3 |  |  | 5584 | -0.142 | -0.6691 | Yes |
| 123 | CENPW |  |  | 5585 | -0.142 | -0.6670 | Yes |
| 124 | LIMA1 |  |  | 5593 | -0.144 | -0.6661 | Yes |
| 125 | KNSTRN |  |  | 5610 | -0.146 | -0.6666 | Yes |
| 126 | SLC25A5 |  |  | 5628 | -0.148 | -0.6673 | Yes |
| 127 | CDCA8 |  |  | 5642 | -0.151 | -0.6672 | Yes |
| 128 | KIFC1 |  |  | 5645 | -0.152 | -0.6653 | Yes |
| 129 | CCDC34 |  |  | 5647 | -0.152 | -0.6632 | Yes |
| 130 | CLIC1 |  |  | 5664 | -0.154 | -0.6636 | Yes |
| 131 | LSM4 |  |  | 5673 | -0.155 | -0.6627 | Yes |
| 132 | PARPBP |  |  | 5682 | -0.157 | -0.6617 | Yes |
| 133 | TTK |  |  | 5684 | -0.157 | -0.6595 | Yes |
| 134 | TUBG1 |  |  | 5690 | -0.158 | -0.6580 | Yes |
| 135 | MPHOSPH9 |  |  | 5706 | -0.161 | -0.6582 | Yes |
| 136 | CACYBP |  |  | 5713 | -0.162 | -0.6568 | Yes |
| 137 | NUCKS1 |  |  | 5724 | -0.166 | -0.6560 | Yes |
| 138 | HNRNPH3 |  |  | 5737 | -0.169 | -0.6555 | Yes |
| 139 | GPSM2 |  |  | 5739 | -0.169 | -0.6532 | Yes |
| 140 | REEP4 |  |  | 5749 | -0.172 | -0.6521 | Yes |
| 141 | EXOSC8 |  |  | 5751 | -0.173 | -0.6498 | Yes |
| 142 | NCAPD2 |  |  | 5778 | -0.179 | -0.6515 | Yes |
| 143 | MALAT1 |  |  | 5783 | -0.179 | -0.6495 | Yes |
| 144 | NOVA1 |  |  | 5789 | -0.181 | -0.6476 | Yes |
| 145 | TRIP13 |  |  | 5792 | -0.181 | -0.6453 | Yes |
| 146 | PTPRZ1 |  |  | 5811 | -0.186 | -0.6456 | Yes |
| 147 | SERPINE1 |  |  | 5812 | -0.186 | -0.6428 | Yes |
| 148 | MIS18A |  |  | 5816 | -0.187 | -0.6406 | Yes |
| 149 | PSMC3 |  |  | 5831 | -0.190 | -0.6401 | Yes |
| 150 | CMC2 |  |  | 5834 | -0.191 | -0.6376 | Yes |
| 151 | PRC1 |  |  | 5835 | -0.192 | -0.6348 | Yes |
| 152 | PLK1 |  |  | 5836 | -0.192 | -0.6320 | Yes |
| 153 | PTMA |  |  | 5852 | -0.195 | -0.6316 | Yes |
| 154 | IGFBP7 |  |  | 5868 | -0.199 | -0.6312 | Yes |
| 155 | CKAP2 |  |  | 5879 | -0.201 | -0.6299 | Yes |
| 156 | SAE1 |  |  | 5888 | -0.203 | -0.6282 | Yes |
| 157 | ANP32B |  |  | 5890 | -0.204 | -0.6254 | Yes |
| 158 | RBMX |  |  | 5892 | -0.205 | -0.6225 | Yes |
| 159 | DIAPH3 |  |  | 5893 | -0.205 | -0.6195 | Yes |
| 160 | C12orf75 |  |  | 5896 | -0.206 | -0.6168 | Yes |
| 161 | SPAG5 |  |  | 5899 | -0.207 | -0.6141 | Yes |
| 162 | BUB1B |  |  | 5901 | -0.207 | -0.6112 | Yes |
| 163 | SNRPB |  |  | 5908 | -0.209 | -0.6091 | Yes |
| 164 | SNRPD1 |  |  | 5911 | -0.210 | -0.6063 | Yes |
| 165 | PTGES3 |  |  | 5912 | -0.210 | -0.6032 | Yes |
| 166 | PRR11 |  |  | 5926 | -0.214 | -0.6022 | Yes |
| 167 | LSM5 |  |  | 5932 | -0.216 | -0.5999 | Yes |
| 168 | RANBP1 |  |  | 5939 | -0.218 | -0.5977 | Yes |
| 169 | TXNDC12 |  |  | 5947 | -0.220 | -0.5956 | Yes |
| 170 | RACGAP1 |  |  | 5953 | -0.223 | -0.5932 | Yes |
| 171 | CENPA |  |  | 5960 | -0.226 | -0.5908 | Yes |
| 172 | TMEM237 |  |  | 5962 | -0.226 | -0.5877 | Yes |
| 173 | SRSF3 |  |  | 5969 | -0.227 | -0.5853 | Yes |
| 174 | RTKN2 |  |  | 5977 | -0.229 | -0.5831 | Yes |
| 175 | TMEM106C |  |  | 5979 | -0.230 | -0.5799 | Yes |
| 176 | KIF18A |  |  | 5980 | -0.230 | -0.5765 | Yes |
| 177 | TROAP |  |  | 5983 | -0.232 | -0.5734 | Yes |
| 178 | MZT1 |  |  | 5989 | -0.235 | -0.5708 | Yes |
| 179 | RAN |  |  | 5990 | -0.235 | -0.5673 | Yes |
| 180 | STMN1 |  |  | 5993 | -0.236 | -0.5641 | Yes |
| 181 | KIF2C |  |  | 5996 | -0.237 | -0.5610 | Yes |
| 182 | SPC24 |  |  | 6000 | -0.238 | -0.5580 | Yes |
| 183 | KIF14 |  |  | 6006 | -0.240 | -0.5553 | Yes |
| 184 | POC1A |  |  | 6010 | -0.241 | -0.5522 | Yes |
| 185 | RPA3 |  |  | 6014 | -0.244 | -0.5491 | Yes |
| 186 | SFPQ |  |  | 6022 | -0.247 | -0.5466 | Yes |
| 187 | AURKA |  |  | 6026 | -0.248 | -0.5435 | Yes |
| 188 | HNRNPDL |  |  | 6028 | -0.251 | -0.5399 | Yes |
| 189 | HNRNPA2B1 |  |  | 6029 | -0.251 | -0.5362 | Yes |
| 190 | DEPDC1 |  |  | 6030 | -0.251 | -0.5325 | Yes |
| 191 | MXD3 |  |  | 6042 | -0.265 | -0.5305 | Yes |
| 192 | BUB1 |  |  | 6045 | -0.266 | -0.5269 | Yes |
| 193 | HMGB1 |  |  | 6047 | -0.267 | -0.5231 | Yes |
| 194 | HJURP |  |  | 6055 | -0.271 | -0.5203 | Yes |
| 195 | LDHA |  |  | 6057 | -0.272 | -0.5164 | Yes |
| 196 | HSP90AA1 |  |  | 6062 | -0.275 | -0.5130 | Yes |
| 197 | ARHGAP11A |  |  | 6067 | -0.277 | -0.5096 | Yes |
| 198 | FOXM1 |  |  | 6070 | -0.279 | -0.5058 | Yes |
| 199 | SHCBP1 |  |  | 6071 | -0.279 | -0.5017 | Yes |
| 200 | SPC25 |  |  | 6072 | -0.279 | -0.4976 | Yes |
| 201 | CEP55 |  |  | 6076 | -0.281 | -0.4939 | Yes |
| 202 | GMNN |  |  | 6080 | -0.285 | -0.4902 | Yes |
| 203 | FBXO5 |  |  | 6084 | -0.287 | -0.4865 | Yes |
| 204 | KIF22 |  |  | 6089 | -0.290 | -0.4829 | Yes |
| 205 | CENPN |  |  | 6090 | -0.291 | -0.4786 | Yes |
| 206 | DEK |  |  | 6092 | -0.292 | -0.4745 | Yes |
| 207 | NUF2 |  |  | 6093 | -0.293 | -0.4701 | Yes |
| 208 | CCNA2 |  |  | 6100 | -0.297 | -0.4668 | Yes |
| 209 | DCBLD2 |  |  | 6104 | -0.300 | -0.4628 | Yes |
| 210 | LMNA |  |  | 6105 | -0.300 | -0.4584 | Yes |
| 211 | PIF1 |  |  | 6106 | -0.301 | -0.4540 | Yes |
| 212 | CKAP2L |  |  | 6108 | -0.303 | -0.4496 | Yes |
| 213 | KIF11 |  |  | 6109 | -0.304 | -0.4452 | Yes |
| 214 | CCT5 |  |  | 6116 | -0.312 | -0.4416 | Yes |
| 215 | MELK |  |  | 6117 | -0.312 | -0.4369 | Yes |
| 216 | LBR |  |  | 6118 | -0.312 | -0.4323 | Yes |
| 217 | MIS18BP1 |  |  | 6122 | -0.316 | -0.4282 | Yes |
| 218 | PHF19 |  |  | 6129 | -0.322 | -0.4244 | Yes |
| 219 | HMGA1 |  |  | 6132 | -0.324 | -0.4200 | Yes |
| 220 | LMNB1 |  |  | 6134 | -0.325 | -0.4154 | Yes |
| 221 | CDCA2 |  |  | 6135 | -0.326 | -0.4106 | Yes |
| 222 | CENPH |  |  | 6136 | -0.327 | -0.4057 | Yes |
| 223 | DDX39A |  |  | 6137 | -0.328 | -0.4009 | Yes |
| 224 | TACC3 |  |  | 6138 | -0.328 | -0.3960 | Yes |
| 225 | CCNB2 |  |  | 6140 | -0.329 | -0.3913 | Yes |
| 226 | NDC80 |  |  | 6142 | -0.330 | -0.3866 | Yes |
| 227 | AURKB |  |  | 6143 | -0.332 | -0.3817 | Yes |
| 228 | TK1 |  |  | 6144 | -0.334 | -0.3768 | Yes |
| 229 | ARL6IP1 |  |  | 6145 | -0.336 | -0.3719 | Yes |
| 230 | NUDT1 |  |  | 6146 | -0.338 | -0.3669 | Yes |
| 231 | HMMR |  |  | 6148 | -0.340 | -0.3620 | Yes |
| 232 | CNIH4 |  |  | 6151 | -0.343 | -0.3573 | Yes |
| 233 | CDC20 |  |  | 6154 | -0.344 | -0.3526 | Yes |
| 234 | DLGAP5 |  |  | 6156 | -0.345 | -0.3476 | Yes |
| 235 | CDKN3 |  |  | 6160 | -0.349 | -0.3430 | Yes |
| 236 | KIF23 |  |  | 6162 | -0.351 | -0.3380 | Yes |
| 237 | HMGN2 |  |  | 6163 | -0.351 | -0.3328 | Yes |
| 238 | KIF4A |  |  | 6164 | -0.355 | -0.3275 | Yes |
| 239 | CDCA3 |  |  | 6165 | -0.355 | -0.3223 | Yes |
| 240 | ANP32E |  |  | 6168 | -0.357 | -0.3174 | Yes |
| 241 | CENPK |  |  | 6169 | -0.363 | -0.3120 | Yes |
| 242 | KIF20B |  |  | 6172 | -0.373 | -0.3068 | Yes |
| 243 | DTYMK |  |  | 6177 | -0.381 | -0.3019 | Yes |
| 244 | ECT2 |  |  | 6178 | -0.382 | -0.2963 | Yes |
| 245 | CENPM |  |  | 6179 | -0.386 | -0.2906 | Yes |
| 246 | DBF4 |  |  | 6180 | -0.387 | -0.2848 | Yes |
| 247 | IGFBP3 |  |  | 6184 | -0.397 | -0.2795 | Yes |
| 248 | ZWINT |  |  | 6195 | -0.425 | -0.2749 | Yes |
| 249 | TUBB4B |  |  | 6196 | -0.431 | -0.2685 | Yes |
| 250 | RRM2 |  |  | 6199 | -0.438 | -0.2624 | Yes |
| 251 | ESCO2 |  |  | 6206 | -0.463 | -0.2566 | Yes |
| 252 | PBK |  |  | 6207 | -0.465 | -0.2497 | Yes |
| 253 | BIRC5 |  |  | 6214 | -0.484 | -0.2436 | Yes |
| 254 | ANLN |  |  | 6215 | -0.484 | -0.2364 | Yes |
| 255 | GTSE1 |  |  | 6217 | -0.490 | -0.2294 | Yes |
| 256 | SMC4 |  |  | 6219 | -0.490 | -0.2223 | Yes |
| 257 | NUSAP1 |  |  | 6223 | -0.507 | -0.2153 | Yes |
| 258 | CENPE |  |  | 6226 | -0.511 | -0.2081 | Yes |
| 259 | NCAPG |  |  | 6227 | -0.513 | -0.2005 | Yes |
| 260 | SMC2 |  |  | 6229 | -0.518 | -0.1931 | Yes |
| 261 | GGH |  |  | 6232 | -0.522 | -0.1857 | Yes |
| 262 | TMPO |  |  | 6233 | -0.522 | -0.1780 | Yes |
| 263 | CKS1B |  |  | 6234 | -0.523 | -0.1703 | Yes |
| 264 | KPNA2 |  |  | 6236 | -0.543 | -0.1624 | Yes |
| 265 | H2AFX |  |  | 6237 | -0.576 | -0.1539 | Yes |
| 266 | CKS2 |  |  | 6239 | -0.585 | -0.1455 | Yes |
| 267 | UBE2T |  |  | 6240 | -0.593 | -0.1367 | Yes |
| 268 | CCNB1 |  |  | 6242 | -0.606 | -0.1279 | Yes |
| 269 | UBE2S |  |  | 6244 | -0.617 | -0.1190 | Yes |
| 270 | PTTG1 |  |  | 6245 | -0.622 | -0.1098 | Yes |
| 271 | CDK1 |  |  | 6246 | -0.627 | -0.1006 | Yes |
| 272 | MAD2L1 |  |  | 6247 | -0.628 | -0.0913 | Yes |
| 273 | H2AFZ |  |  | 6250 | -0.651 | -0.0820 | Yes |
| 274 | TPX2 |  |  | 6253 | -0.667 | -0.0725 | Yes |
| 275 | ASPM |  |  | 6254 | -0.680 | -0.0625 | Yes |
| 276 | UBE2C |  |  | 6255 | -0.750 | -0.0514 | Yes |
| 277 | HMGB2 |  |  | 6258 | -0.766 | -0.0405 | Yes |
| 278 | TOP2A |  |  | 6260 | -0.814 | -0.0286 | Yes |
| 279 | MKI67 |  |  | 6261 | -0.882 | -0.0156 | Yes |
| 280 | CENPF |  |  | 6263 | -1.068 | 0.0000 | Yes |
Table: GSEA details [plain text format]

  

Fig 2: FELDMAN\_2020\_G2-M: Random ES distribution      
 Gene set null distribution of ES for **FELDMAN\_2020\_G2-M**
